# Functionalized Antimicrobial Nanofibers: Design Criteria and Recent Advances

**DOI:** 10.3390/jfb12040059

**Published:** 2021-10-28

**Authors:** Nazirah Hamdan, Alisa Yamin, Shafida Abd Hamid, Wan Khartini Wan Abdul Khodir, Vincenzo Guarino

**Affiliations:** 1 Department of Chemistry, Kulliyyah of Science, International Islamic University Malaysia Kuantan Campus, Bandar Indera Mahkota, Kuantan 25200, Malaysia; nazirahhamdan11@gmail.com (N.H.); alisayamin01@gmail.com (A.Y.); shafida@iium.edu.my (S.A.H.); 2 SYNTOF, Kulliyyah of Science, International Islamic University Malaysia Kuantan Campus, Bandar Indera Mahkota, Kuantan 25200, Malaysia; 3 Institute of Polymers, Composites and Biomaterials, National Research Council of Italy, Mostra d’Oltremare Pad.20, V.le J.F.Kennedy 54, 80125 Naples, Italy

**Keywords:** nanofibers, electrospinning, surface functionalization, antimicrobial resistance, non-antibiotic treatments

## Abstract

The rise of antibiotic resistance has become a major threat to human health and it is spreading globally. It can cause common infectious diseases to be difficult to treat and leads to higher medical costs and increased mortality. Hence, multifunctional polymeric nanofibers with distinctive structures and unique physiochemical properties have emerged as a neo-tool to target biofilm and overcome deadly bacterial infections. This review emphasizes electrospun nanofibers’ design criteria and properties that can be utilized to enhance their therapeutic activity for antimicrobial therapy. Also, we present recent progress in designing the surface functionalization of antimicrobial nanofibers with non-antibiotic agents for effective antibacterial therapy. Lastly, we discuss the future trends and remaining challenges for polymeric nanofibers.

## 1. Introduction

In recent decades, the inception of multidrug-resistant (MDR) bacteria or “superbugs” has become a global threat due to the resistance of bacteria to antibiotics. The treatment of MDR bacteria with ineffective antibiotics has formed new resistances that have spread remarkably across continents through the environment, people, and animals. The 2019 Antibiotic Resistance Threats report from the Centers for Disease Control and Prevention (CDC) classified a few multidrug-resistant bacteria and fungi based on their threat levels to human health, and reported that in the US, more than 2.8 million antibiotic-resistant infections occur yearly, resulting in the death of more than 35,000 people. In addition, 223,900 cases of *Clostridioides difficile* arose in 2017 and caused mortality in 12,800 people [1]. US hospitals also repot around 40–60% of *Staphylococcus aureus* strains collected are resistant to methicillin and even vancomycin and carbapenems [2]. The increase of the morbidity and mortality statistics worldwide challenges healthcare institutions and the community to overcome the issue of the misuse of antibiotics, and inadequate infection prevention and treatment had increased the number of MDR bacteria to develop and spread alarmingly [3,4,5]. Notably, bacterial infection cases are primarily responsible for the excess health costs in the US. 

Antibiotics act on bacteria by inhibiting their cell walls and interfering with DNA, RNA, or essential proteins. However, bacteria innately have the ability to alter their structural properties and characteristics to reduce the efficacy of antibiotics [6,7,8,9,10]. Bacterial cells can also adapt to external stimuli by altering their gene and protein expressions [11]. Antibiotic resistance in bacteria will increase as the number of multidrug-resistance strains continues to grow. This phenomenon leads to an urgent need to discover non-antibiotic routes as alternative antimicrobial therapies against these highly resistant bacteria. With the evolution of nanotechnology, nanostructured materials are gaining interest and attention in biomedical applications. Electrospun polymeric nanofibers exhibit unique physicochemical properties such as size, shape, and surface chemistry that influence their therapeutic activity and thus offer flexibility that makes them easily tailored for antimicrobial therapy [12,13,14,15,16,17,18]. 

There is increasing interest in utilizing polymeric nanofibers with a drug cargo of antibiotics in killing bacteria. Most studies have only focused on intrinsic structure and tunable structure, components, and properties of nanofibers which enable the generation of drug-loaded nanofibers with a sustained release pattern for drug delivery application [19,20,21]. Nanofibers can fight against bacteria with their beneficial topography features. However, there is still a lack of understanding on how these nanofibers can kill bacteria. In this review, we mainly discuss the influence of nanofiber properties and their bactericidal interactions, as well as the properties of nanofibers, including their morphologies, surface charge, wettability, and functionalization to be considered to ensure antimicrobial efficiency. We also highlight topographical features using different surface functionalization-based approaches with antimicrobial agents such as metals, metal oxides, metal nanoparticles, graphene oxide, peptides, and natural extracts [12,22,23,24,25,26] to optimize their therapeutic activity against the multidrug-resistance and biofilm of bacteria. We hope this review will provide a guideline to design effective, functionalized antimicrobial nanofibers for a wide range of biomedical applications. From the points mentioned above, nanofibers are a promising toolkit for the non-antibiotic treatment for bacterial infection.

## 2. Polymeric Nanofibers and Electrospun Scaffolds

In recent years, electrospun nanofiber scaffolds have been demonstrated to be effective nano-scale therapeutic devices, as their physicochemical properties can be tailored to several applications requiring necessary antimicrobial capabilities [27,28,29]. In particular, nanofibrous structures have several intrinsic properties which make them peculiarly functional to design for antimicrobial applications [19,30,31,32,33,34]. Ideally, fiber diameters at the nanometric scale make their structure suitable to bio-mimic the natural extracellular matrix (ECM) of tissue, thus providing a friendly environment for the regeneration of the target site and facilitating repair mechanisms [33,34,35]. In addition to topographical fibers resembling native ECM architecture, they can also influence cell migration, adhesion, differentiation, and regeneration [36,37,38,39,40,41].

Due to their small size, nanofibers possess a very large surface area-to-volume ratio along with interconnectivity and microscale interstitial space, rendering them more effective than their bulk form. The high surface area of nanofibers can promote the hemostasis of injured tissues and fluid absorption [42,43,44] and they are also effective at delivering a drug cargo to the target site [45,46,47,48,49]. For example, Giram et al., (2018) fabricated Eudragit L-100 nanofibers to encapsulate moxifloxacin hydrochloride for a fast drug delivery system. The cylindrically shaped nanofibers were reported able to encapsulate 95–98% of the drug at 1–5% *w*/*w* concentration. The antibiotic-loaded nanofibers also showed good antimicrobial activity against both *Escherichia coli* and *S. aureus* [50].

The performance of nanofibers is influenced highly by their porosity (60–90%) [51,52,53], which allows high surface and wetting permeability, which in turn affect cell proliferation, vascularization and mechanical stability [54,55,56,57]. In addition, the interconnected nanopores on nanofibers’ fractal structure, along with their excellent surface energy, surface reactivity, and high thermal and electric conductivities could prevent the infiltration of microbes and discourage cell ingrowth [58]. All of the above reasons make electrospun nanofibers potentially useful as antimicrobial materials [18,59]. The surface of nanofibers can be modified and functionally used as a conformal surface coating to provide a controlled interaction with microorganisms [60]. Coating the nanofiber surfaces with antibacterial substrates can further enhance the nanofibers’ topography to encourage specific interactions between bacteria and nanofibers [60,61]. Table 1 summarizes the basic fundamental properties of nanofiber scaffolds for antimicrobial applications.

Natural and synthetic polymers are widely used to fabricate nanofiber matrices due to their processability, biocompatibility, and biodegradability [77,78,79,80]. The promising polymer used for the development of electrospun nanofibers for antimicrobial application is summarized in Table 2. Natural polymers are derived from proteins and carbohydrates such as cellulose, chitosan, gelatin, elastin, and polypeptides [81,82,83,84] Chitosan, a versatile hydrophilic polysaccharide derived from chitin, is frequently used to develop nanofibers. It exhibits good antimicrobial activity against several strains of microbes such as *S. aureus*, *E. coli*, *Listeria innocua* and *Salmonella typhymurium* [85,86]. In contrast to natural polymers, the simpler chemical structure of synthetic polymers brings ease of processability and provides nanofibers with good mechanical properties [83,87]. The common synthetic polymers used are poly(ε-caprolactone) (PCL), poly-lactide (PLA), poly-glycolide (PGA), polyvinyl alcohol (PVA), and polydimethylsiloxane (PDMS) [88,89,90,91]. Natural and synthetic polymers have their advantages and disadvantages [92,93,94,95,96,97], and researchers often combine them using electrospinning techniques to achieve nanofibers with better physico-chemical properties [69,98,99]. The combination of natural and synthetic polymers can be achieved through multiple strategies. In our previous study, the blending of PVA and chitosan provided better thermal stability for nanofibers to encapsulate gentamicin for controlled release of up to 72 h [96]. Meanwhile, Guarino et al., (2017) fabricated PCL nanofibers and functionalized them with chitosan as a reservoir for amoxicillin trihydrate to improve the entrapment and release of antibiotics for targeted antimicrobial applications [66].

The electrospinning technique is a straightforward process to fabricate polymeric nanostructures and thus offers the versatility of structure, morphology, and spatial distribution of electrospun nanofibers to achieve specific mechanical properties [23,105,106,107]. The technique has been used to fit the purpose of various applications from small-scale basic research applications to large scales of nanofibers relevant for industrial purposes [71,108,109]. Comprehensive reviews on the theory of the electrospinning process are already available [71,110,111,112] and thus, here we provide a brief overview on how the electrospinning process works.

A typical electrospinning setup consists of a high-voltage power supply, a ground collector, a syringe pump, and a syringe with a capillary needle. The polymer solution is loaded into a syringe attached to the needle at a controlled flow rate. The repulsive electrical force is applied to overcome the surface tension of the polymer solution, resulting in the formation of a Taylor cone. The polymer solution will stretch and evaporate, and the fibers will be deposited on the metal-conductive rotating ground collector [75,113,114,115]. Generally, electrospun nanofibers can be oriented as aligned or non-aligned (random) structures. Aligned nanofibers can be prepared using a rotating collector [26,116,117,118] while non-aligned nanofibers use only a simple conductive metal plate [119,120,121]. Aligned nanofibers have been reported to closely mimic the native extracellular matrix structure, thus promoting cell migration or proliferation [74,122]. While aligned nanofibers provide better mechanical strength and allow better incorporation of therapeutic agents [118,123,124,125], non-aligned nanofibers are easier to fabricate and have a higher entrapment capacity to incorporate therapeutic agents or to enable sustained release in a specific site of action [50,67,103,126]. In terms of bacterial attachment, the antimicrobial effect of the nanofibers was found to be independent of their alignment, as there is no significant difference between antimicrobial activity for both orientations [117]. 

The unique characteristics of electrospun nanofibers, such as high-surface-to-volume ratio, controllable fibers orientation and diameters, high porosity, and modulated surface roughness, are greatly influenced by the electrospinning process. Thus, the morphological features of electrospun nanofibers can be altered by tuning parameters such as the polymer solution properties (concentration, viscosity, conductivity, dielectric constant, and surface tension) or processing parameters such as applied voltage, solution flow rate, tip-to-collector distance, and collector speed [114,127,128]. In order to produce uniform and bead-less nanofibers, the optimum polymer concentration and viscosity are required to allow adequate chain entanglement and surface tension [92,99]. A low voltage applied during electrospinning may result in beads and small-diameter nanofibers, while a high applied voltage may result in thick and non-homogeneous nanofibers [59,99,129]. Apart from that, the solution flow rate also affects the morphology of nanofibers [129]. A relatively low flow rate produces nanofibers with beads and broken strands. At the same time, too high a flow rate produces droplets due to the higher velocity of the polymer solution being charged and ejected from the tip [99,129,130]. The nanofibers’ diameter can decrease as the tip-to-collector distance increases, allowing complete solvent evaporation at an optimal distance [129,131]. All of these parameters are interrelated, and thus it is essential to optimize and tune each parameter to obtain nanofibers with specific morphological characteristics for the desired antimicrobial applications. 

**Figure 1:**
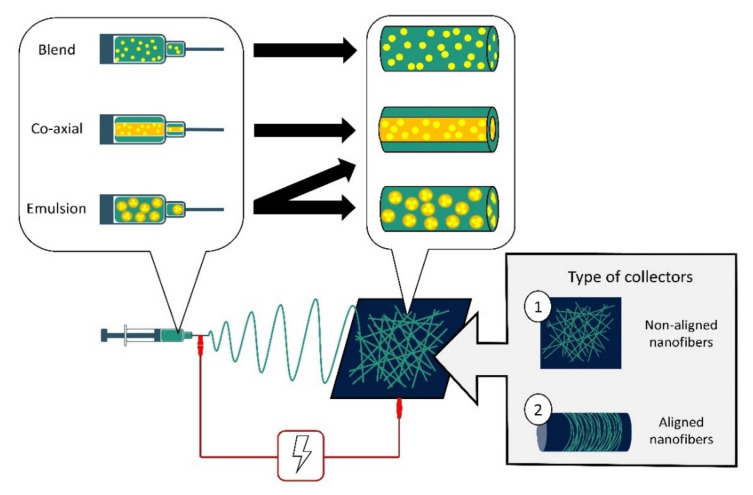
Schematic diagram of an electrospinning setup and variations of electrospinning techniques. Therapeutic agents can be incorporated in nanofibers via blend, co-axial and emulsion electrospinning. Blend solution electrospinning results in the therapeutic agents being well distributed in the nanofibers. Co-axial electrospinning allows the therapeutic agents to be timely delivered from core-shell nanofibers. Emulsion electrospinning may form two types of nanofibers—either the emulsion (consisting of the therapeutic agents) coalesces to form a core similar to the fibers expected of the co-axial electrospinning technique, or the emulsion will disperse varyingly in the nanofibers. Two types of collectors are mainly used in electrospinning: (1) flat plate collectors fabricating non-aligned nanofibers and (2) rotating drum collectors fabricating aligned nanofibers.

Nanofibers have been used as a drug cargo delivery vehicle for therapeutic agents such as antibiotics, metal nanoparticles, carbon materials, peptides, and natural extracts [100]. These therapeutic agents can be directly incorporated into the nanofibers’ matrices using several approaches such as blend electrospinning, emulsion electrospinning and co-axial electrospinning [17,101,132,133]. Different methods of electrospinning will produce nanofibers with different morphologies (Figure 1). In blend electrospinning, the therapeutic agent is dissolved in the polymer solution before electrospinning. Thus, it is well distributed throughout the nanofibers [130]. Meanwhile, emulsion electrospinning involves two immiscible phases of polymers and a therapeutic agent, whereby the agent can be encapsulated throughout the nanofibers matrix or encapsulated in the core-shell nanofibers [131,132,133]. Co-axial electrospinning uses two nozzles containing the polymer solution and the therapeutic agent separately, to produce a core-shell structure in the nanofibers [134,135,136,137]. Usually, the polymer matrix will provide the outer core, while the therapeutic agent is incorporated in the inner core of the nanofibers [27,138]. Recently, researchers have shown an effort to develop 3D electrospun polymeric nanofiber scaffolds using several combinations of techniques such as co-axial electrospinning with add-on techniques such as electrospraying, 3D printing, gas foaming, freeze-drying, and centrifugal electrospinning, to obtain multifunctional structures. A 3D scaffolds with a well-defined spatial organization of the therapeutic agent in the membranes could offer spatiotemporal release [139,140].

## 3. Nanofiber Action towards Bacteria

**Figure 2:**
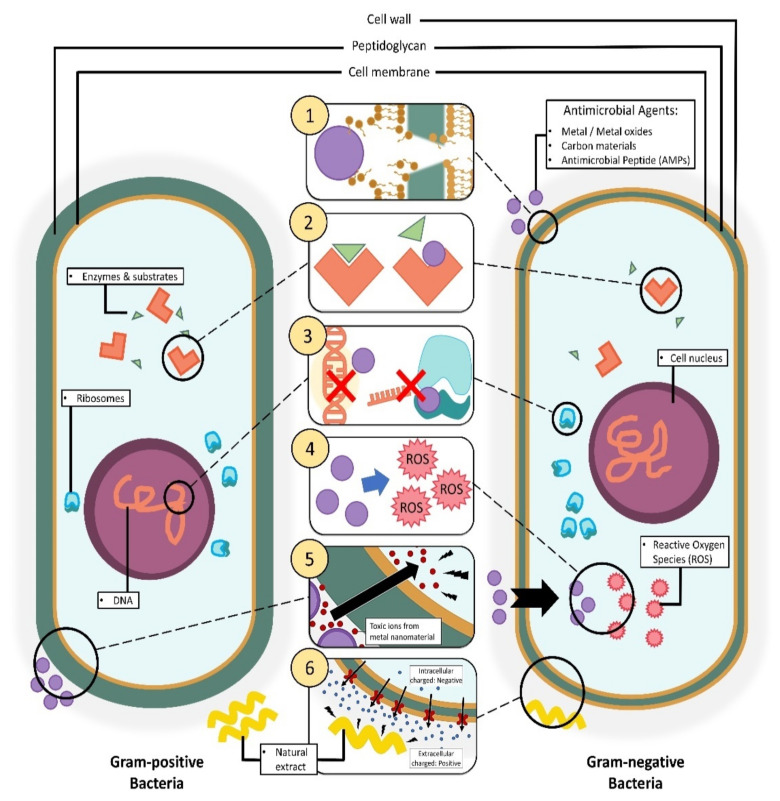
Illustration of different mechanisms of action by antimicrobial agents incorporated in nanofibers on bacteria cells via: (1) disruption of the cell membrane/cell wall. (2) Inhibition of cellular metabolic pathways. (3) Inhibition of DNA and gene expression. (4) Instigation of cellular oxidative stress. (5) Metal-based nanomaterial toxification. (6) Cellular hyperpolarization.

In the initial stage of the infectious process, gram-positive microbes such as *S. aureus* and *Streptococcus pyogenes* are the dominant organisms involved, while gram-negative organisms like *E. coli* and *P. aeruginosa* are only found in later stages of the process, i.e., when a chronic wound has developed [141,142,143]. In order to kill bacteria, it is imperative to understand the bacteria structures as the cell wall of the bacteria is the primary barrier for the penetration of antimicrobial agents. Gram-positive bacteria have a cell wall made of a thick and rigid peptidoglycan layer (>10 layers) with polymeric teichoic acids and a cytoplasmic membrane. The teichoic acid polymeric chains have a phosphate group that provides a negative charge to bacterial surfaces and serves as a binding site for the divalent cations in the solution [144,145,146,147]. On the other hand, gram-negative bacteria have a thin cytoplasmic membrane, thin peptidoglycan layer, and lipopolysaccharides, which can reduce the penetration ability of antimicrobial agents (Figure 2) [148,149,150]. This is one reason why gram-negative bacteria are harder to penetrate compared to gram-positive bacteria. The bacterial cell wall is vital for osmotic regulation, heat tolerance, phage-binding, and cell-shape determination [146,147,151]. Further adhesion of antimicrobial therapeutics onto the bacteria can improve their penetration ability for efficient delivery [145,152,153,154]. 

The inherent antimicrobial activity and mechanism of nanofibers alone have not been widely explored. Nanofibers’ most well-documented antimicrobial activity revolves only around electrospun chitosan nanofibers—chitosan being a natural polymer with antimicrobial properties [82,147,149]. The protonated amino groups of the chitosan nanofibers were implied to be responsible for the antimicrobial activity against *S. aureus*, *E. coli*, *L. innocua*, and *S. typhymurium* [82]. An antimicrobial test of chitosan nanofibers was conducted against *Clostridium difficile* isolates with tetracycline and chloramphenicol resistance genes. The successful bacterial inhibition activity suggested that protein synthesis disruption is not the mechanism of the antibacterial action of chitosan nanofibers [149]. This implies that nanofibers alone are not enough to inhibit bacteria. Therefore, functionalization with antimicrobial agents is required to improve their bactericidal effect. 

Functionalized nanofibers can express various bactericidal pathways depending on the core material used, the morphology of the material, and the surface chemistry of the scaffold [150,155]. The enhanced cell membrane penetration ability of functionalized nanofibers and their potential to modulate cellular interaction make them a viable candidate for treating bacterial infections [151,156]. The exact mechanism of bactericidal pathways involving functionalized nanofibers is unknown, and the assumptions from previous research studies differ as a function of the additional components used [148,157]. 

The bactericidal effects of nanofibers depend on their size, diameter, shape, and surface chemistry. Three-dimensional nanofiber scaffolds with hierarchical structures as small as a few microns to a few hundred nanometers provide a high surface area and thus enhanced therapeutic efficacy against bacterial infection [74,106]. A recent study by Abrigo et al., (2015) showed that smaller size nanofibers ranging from 300–1000 nm, close to the bacteria’s size, can induce conformational changes of rod shape bacteria, which would lead to cell lysis. On the other hand, when the diameter is larger than the size of bacteria (>5000 nm), they tend to adhere onto the surface and proliferate along the nanofibers [62]. Therefore, nanofibers with small diameters are preferable, as they can alter the bacteria’s conformation and thus increase their susceptibility.

The bacterial adhesion and attachment surface interaction is essential for biofilm control, and is influenced by the surface chemistry such as the surface charge, roughness, topography, and wettability [158,159,160]. To further understand the influence of such material properties, we further discuss the interaction between bacteria and different types of nanofiber surfaces. Most bacteria cells surfaces are negatively charged, and due to the electrostatic force, they are highly attracted to a positively charged surface for bacterial adhesion and attachment [161,162,163,164]. In contrast, a negatively charged surface of material is needed as a resistance mechanism to bacterial adhesion. Surfaces with certain cationic groups such as quaternary ammonium and polyethyleneimine have antimicrobial activity and thus can kill the bacteria cells. For instance, MRSA is highly attracted to the positively charged poly-(lactic-co-glycolic acid) (PLGA) functionalized with polyethyleneimine (PLGA-PEI) surface, as compared to the negatively charged PLGA [161]. This implies that biocidal active molecules incorporated into nanofibers with positively charged moieties can effectively be released to bacterial cells upon contact with the bacterial cell wall. Another study found that flat biofilms developed on the positively charged surface of poly(2-(methacryloyloxy)-ethyl trimethyl ammonium chloride had higher binding affinity compared to the negatively charged poly (3-sulphopropylmethacrylate) [165]. It was suggested that the negatively charged polymer surface efficiently repelled bacterial adhesion and prevented biofilm formation. It was also revealed that E. coli attachment was higher on a polyethylene-glycidyl methacrylate sheet functionalized with diethylamine (positively charged) compared to sodium sulfite (negatively charged) due to electrostatic repulsion [166]. The viability of *E. coli* cells had also significantly decreased after attachment onto the diethylamine surface, but remained high on sodium sulfite surface [166]. Despite that, it has been reported that highly charged cationic polymers exhibit cytotoxicity to bacteria and human cells, where it can cause agglutination of red blood cells [9,167]. For example, 6-Deoxy-6-(2-aminoethyl) amino chitosan (CS-AEA), a chitosan derivative, demonstrated higher agglutination performance due to the higher amount of protonated amine groups and degree of ionization compared to chitosan [168]. Hence, it is essential to consider tuning the surface charge in balance for a potent antimicrobial application. 

Meanwhile, rough surface nanofibers with large surface areas promote bacterial contact and attachment due to higher bacterial contact are [107,163]. However, surface roughness alone is not enough to attract the bacteria. Ludecke et al., (2016) showed that the number of bacteria attached was found to decrease even though the nanofibers have a rough surface. Their study indicates that the nanofibers also need to have a maximum contact area for the bacteria to adhere to its surface [169]. Increased surface roughness can promote bacterial adhesion and induce mechanical disintegration of the bacteria structure, leading to increase bacterial susceptibility [152,159]. Hence, for an efficient antimicrobial effect, the nanofibers should be designed to have a rough surface with a higher peak or contact area to encourage bacterial attachment and interaction with the nanofibers. 

Surface wettability plays a crucial role in the attachment or detachment of the biofilm from the surface. The surface wettability can be influenced by the degree of hydrophobicity of the nanofibers [170]. Hydrophobic bacteria such as S. aureus has been shown to adhere firmly to hydrophobic surfaces due to their similar chemical characteristic [171,172]. In contrast, a hydrophilic surface can effectively inhibit the adhesion of the bacteria, as the surface bonding between the bacteria and nanofibers is weak [107]. The fabrication of cationic nanofibers using polystyrene and poly(ethylene terephthalate) increased the antimicrobial potency as they are positively charged and highly hydrophobic [173,174,175].

The porosity, which is three-dimensional (3D) holes formed on the nanofibers, can influence bacterial attachment by affecting surface wettability [106]. Porous nanofibers with a large pore diameter ranging from 50-100 nm are more favorable for bacterial attachment than non-porous or porous nanofibers with pore diameter less than 25 nm [73,163].

**Figure 3:**
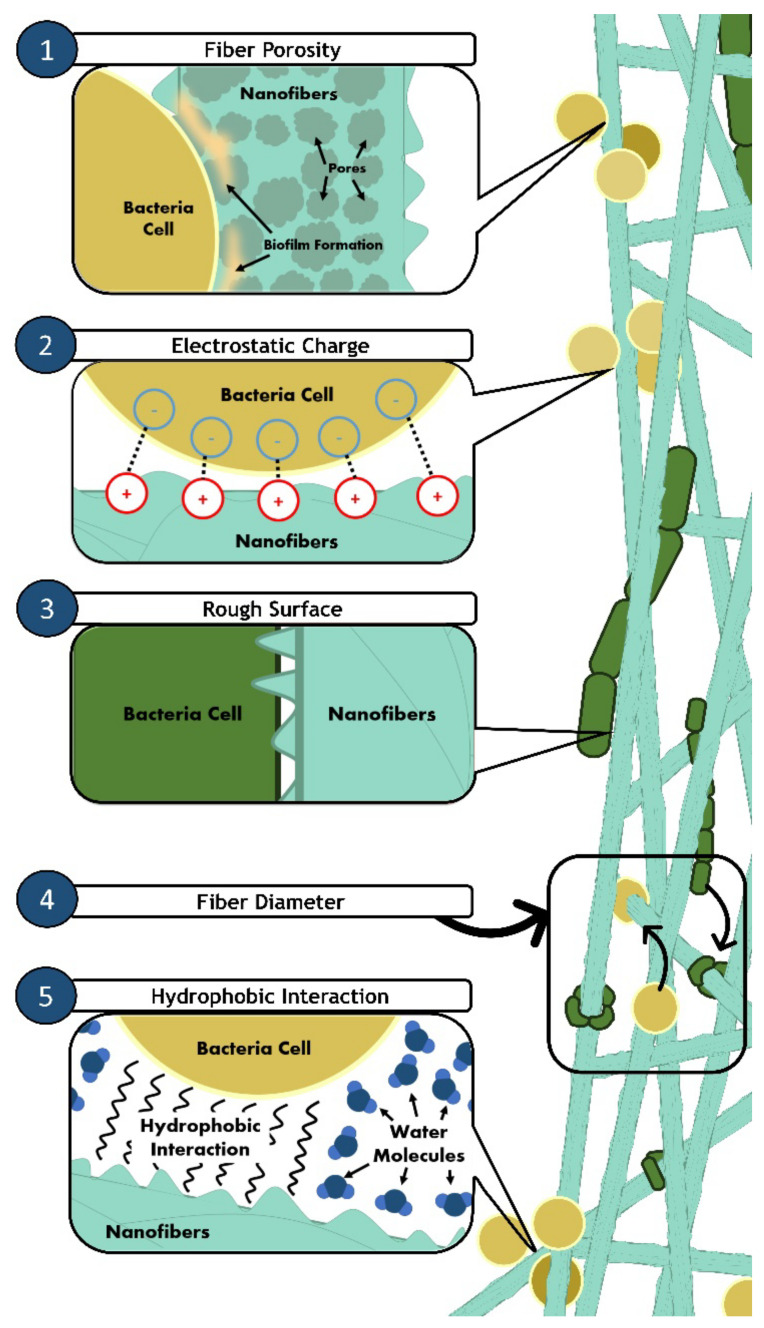
Illustration of bacterial adhesion on nanofibers. Various properties of nanofibers can induce and enhance bacterial attachment. (1) Fiber porosity (nano-sized) allows early biofilm formation, causing bacteria cells to attach easily onto highly porous nanofibers. (2) Nanofibers with a positive surface charge will also attract the negatively charged surface of bacterial cells. (3) Rough surfaces of nanofibers also provide more area of contact for bacteria cells to attach. (4) Thin fiber diameters (smaller than bacteria size) also allow changes in bacterial cells’ conformation. The surface wettability of nanofibers plays a significant role in bacterial adhesion. (5) Hydrophobic bacterial cells will adhere to the surface of hydrophobic nanofibers due to hydrophobic interactions.

To sum up, functionalized nanofibers should be designed to have: (1) small diameters (300–1000 nm), (2) positively charged surfaces, (3) rough surfaces with high surface area, (4) hydrophobic surfaces, and also (5) large pore diameters to ensure better adhesion (Figure 3) to improve bactericidal effects. Despite the lack of a specified mechanism of actions for the nanofibers bactericidal pathway, the evidence of unquestionable bactericidal activity from functionalized nanofibers makes it essential to address the proper design criteria of scaffolds for the fabrication of antimicrobial nanofibers.

## 4. Entrapment of Antimicrobial Agents into Nanofibers: Classification

Various nanomaterials such as metal nanoparticles, nanodots (i.e., carbon nanotubes), nano blades (i.e., graphene sheet) and nano spikes (i.e., cicada wings) showed the effectiveness of the mechano-bactericidal mechanism in penetrating and rupturing bacterial cell walls, eventually causing cell death [152,153,154]. These types of nanomaterials can be incorporated on the surface of nanofibers (which acts as a stable base) to impart biocidal ability, thus developing antimicrobial nanofibers.

### 4.1. Metal, Metal Oxides and Metal Nanoparticles

Metal and metal oxide nanoparticles are known for their ability to penetrate bacterial cells and disrupt cellular activity by generating reactive oxygen species (ROS) such as hydrogen peroxide or superoxide anions. Excessive ROS production will cause severe oxidative stress that will damage the bacterial cellular components, disrupt protein synthesis, inhibit enzymatic action, and cause cell membrane disruption and site-specific DNA damage, ultimately leading to cell lysis [176,177,178]. In recent years, metal nanoparticles such as silver and gold, as well as metal oxide nanoparticles such as zinc oxide, iron oxide, titanium dioxide, and copper oxide have been extensively studied for antimicrobial applications [31,157,176,179,180,181]. Although the bactericidal ability of these metal and metal oxide nanoparticles is well documented, the precise mechanism of action is still unknown. Composite materials involving various metal nanoparticles in nanofibers have been executed extensively in the past, with ample evidence of successful antimicrobial activity [182,183,184,185,186,187].

Metals like silver (Ag), gold (Au), copper (Cu), zinc (Zn) and their corresponding oxides are commonly used to design potent antimicrobial nanomaterials [188]. Among the metals, silver (Ag)-based nanofibers have been studied extensively since Ag ions (Ag+) are known to be toxic to bacteria and microorganisms even at low concentrations [188]. Ag nanoparticles showed good antimicrobial activity against *E. coli*, *S. aureus* and *P. aeruginosa* [189]. Chitosan/polyvinyl alcohol (PVA) nanofibers loaded with silver ion-incorporated hydroxyapatite (HAP) nanoparticles were reported to inhibit the growth of *E. coli* even at low Ag concentrations (0.5% *w*/*v*). The bacteria inhibition zones observed were increased as the concentration of Ag increased due to the increase of metal toxicity [190]. Moon et al., (2021) fabricated 3D cellulose nanofibers decorated with Ag-nanoparticles using the gas foaming technique. The composite design enhanced the nanofibers’ structural and mechanical stability and showed excellent antimicrobial activity against *S. aureus* and *E. coli* [191]. In a study by Li et al., (2013), PVA/chitosan oligosaccharide (PVA/COS)-loaded Ag-nanoparticle nanofibers (PVA/COS/Ag-NP) were shown to inhibit the growth of *S. aureus* and *E. coli* [192]. It is worth noting that even though Ag is toxic to bacteria, it is non-toxic to humans in nanoparticle form [189]. The viability of human fibroblast cells decreased significantly when in contact with PVA/COS loaded AgNO3 nanofibers. At the same time, there was no significant cytotoxicity observed, indicating that Ag in nanoparticles form is non-toxic, biocompatible, and thus, safe for humans. 

Unlike Ag, metal ions like zinc ions, Zn+ are essential for bacteria to regulate several metabolic pathways such as sugar, lipid, and protein degradation [193]. However, an excess amount of Zn can result in protein denaturation and malfunction, as well as enzymatic inactivation, thus increasing bacterial susceptibility [188]. This is illustrated by the incorporation of zinc oxide (ZnO) into chitosan/polyvinyl alcohol (chitosan/PVA/ZnO) nanofibers, which showed higher antibacterial activity against *E. coli*, *P. aeruginosa*, *Bacillus subtilis* and *S. aureus* compared to chitosan/PVA nanofibers. In vivo wound healing analysis also revealed that the chitosan/PVA/ZnO nanofibers accelerated the wound healing of subcutaneous wounds in induced diabetic rabbits [194].

Similar to Zn, copper ions, Cu2+ are also essential for biological processes such as the enzymatic reactions and protein interactions of bacteria. However, excess Cu2+ concentrations may lead to cell membrane and DNA disruptions [195]. The Cu2+ ions released from poly(lactic-co-glycolide)/copper oxide (PLGA/CuO) nanofibers were shown to inhibit the growth of both gram-negative *E. coli* and gram-positive *S. aureus* [126]. The ions are believed to adhere to the protein-containing sulfur in the bacteria cell wall, penetrating the cell membrane and then killing the bacteria through protein disruption and direct membrane damage [126,196]. 

### 4.2. Carbon Materials 

Carbon materials such as carbon dots, fullerene, graphite, graphite oxide, graphene oxide and reduced graphene oxide can cause bactericidal effects towards bacteria via several mechanisms: (1) membrane stress induced by the sharp edges of carbon material nanosheets, which can lead to membrane damage and results in the leakage of RNA and other intracellular electrolytes [197,198,199], (2) cellular oxidative stress which can disrupt bacterial lipid, protein and DNA process, resulting in cell death [200], (3) mechanical destruction of the bacteria cell through cell entrapment, which restricts the nutrients entering the cell and later results in cell lysis [156], and (4) bacterial toxicity [201]. Graphene oxide shows the highest antimicrobial activities, followed by reduced graphene oxide, graphite and graphite oxide [156,201]. However, the antimicrobial activity of these carbon materials is highly dependent on the material concentration, density of functional groups, size, and conductivity [202,203]. Therefore, these criteria can be tailored to increase the antimicrobial effects of carbon-incorporated nanofibers for a practical therapeutic application.

The effect of graphene oxide (GO) size evaluated from GO in bacterial suspension showed that the antimicrobial effect of GO increases as its sheet area increases from 0.01 to 0.65 µm^2^ [202]. This is because a larger GO sheet area has a higher capacity to cover the bacteria cells completely, thus altering the cell morphology and integrity [155]. In contrast, the antimicrobial activity of GO-coated surface membranes increases when the GO sheet area decreases from 0.65 to 0.01 µm^2^. Smaller GO sheets have a higher capacity to induce oxidative stress in bacterial cells, leading to membrane damage and cell death [202]. These findings provide a guideline for researchers to modify the GO size accordingly to suit the intended application. In another study, the incorporation of GO in PCL/gelatin nanofibers reduced the diameter of the nanofibers and was found to inhibit 99% of the growth of *E. coli* and *S. aureus* [64]. The inhibition rate also increased significantly as the concentration of GO was increased [30]. Despite its remarkable antimicrobial activity, there is a rising concern regarding GO cytotoxicity for human cells. A cytotoxicity test of GO on human embryonic kidneys (HEK 293) revealed that GO reduces cell viability and proliferation, and increases oxidative stress, leading to DNA damage even at low concentrations (5 and 10 wt%) [201,204]. However, GO loaded in PCL/gelatin nanofibers showed no cytotoxicity effects towards PC 12 neural cells at 1.5 wt% GO concentrations [63]. In addition, it was also found that 0.3 wt% GO loaded in PVA/collagen nanofibers did not show any cytotoxicity towards keratinocyte cells (HaCaT) and further encouraged rapid healing on a group of wounded mice [63]. These results indicate that the toxicity of GO is dose-dependent. Therefore it is vital to control its concentration within the therapeutic range for human use.

Apart from graphene-based materials, carbon quantum dots or carbon dots (CQDs) have been extensively studied for their antimicrobial activity. Nie et al., (2020) found that synthesized CQDs had generated ROS, leading to cell membrane damage, thus inhibiting *E. coli*, *S. aureus*, *Klebsiella pneumoniae*, and multidrug-resistant *Acinetobacter baumannii* [205]. In another study, multifunctional CQD-embedded electrospun polyacrylonitrile (PAN) nanofibers were found to inhibit the growth of *E. coli*. The small size of the CQDs allowed it to penetrate bacteria cells and disrupt the cell wall [206].

Soccer ball-shaped fullerenes (C_60_) have shown antimicrobial activity against *S. aureus*, *E. coli*, and *Shewanella oneidensis*. Fullerene can inhibit the energy metabolism of the bacteria, impair respiratory action, and induce disruption of the cell membrane [207]. Virovska et al., (2016) fabricated electrospun poly(_L_-lactide) (PLA) nanofibers and simultaneously electrosprayed them with a zinc oxide/fullerene (ZnO/C_60_) hybrid. The fiber mats exhibited excellent antimicrobial activity against *S. aureus* at a low fullerene concentration (0.5 and 1.0% *w*/*w*) [208]. The outstanding antimicrobial activity manifested by carbon materials can be considered as a potent non-antibiotic approach for antimicrobial applications.

### 4.3. Antimicrobial Peptides (AMPs)

Other than metal and carbon materials, nanofiber-loaded antimicrobial peptides have also shown a bactericidal effect. Antimicrobial peptides (AMPs) are positively charged peptides with broad-spectrum antimicrobial activity found in various life forms including humans and microorganism [209]. Since most bacteria are attracted to positively charged particles, AMPs can penetrate the bacteria cell membrane [210], and impair the bacterial cell’s osmotic regulation, inhibiting respiration, causing cell membrane rupture, and inducing rapid cell lysis [209]. This mechanism of action reduces the risk of antimicrobial resistance. Hence, it can be a promising alternative to traditional antibiotics. In addition, AMPs also act as an immunological agent which can stimulate and suppress the immune system in response to bacterial threats [211].

As a part of the innate immune response, antimicrobial peptides have a broad-spectrum activity against bacterial infection and demonstrate a potent therapeutic agent [212,213]. The cationic charge of AMPs can cause electrostatic attraction towards bacteria cells and further exhibit bactericidal mechanisms [214]. Song et al., (2016) prepared surface functionalized silk fibroin (SF) nanofibers to immobilize AMP (CYs-KR12) from human cathelicidin peptide (LL37). They found that the Cys-KR12 immobilized onto SF nanofibers inhibited the growth of *S. aureus*, *Staphylococcus epidermidis*, *E. coli* and *P. aeruginosa*. Interestingly, the antimicrobial activity of CYs-KR12 was maintained after three weeks [215]. The nanofibers also promoted the cell proliferation of keratinocytes and fibroblast cells. Investigation of the immunomodulatory effect of the nanofibers towards TNF-α expression of monocytes (Raw 264.7 cells), which can cause chronic inflammation and prolong wound healing, revealed that the Cys-KR12-immobilized SF nanofibers suppressed TNF-α expression, and thus promotes rapid wound healing. In another study, functionalized poly-(acrylic acid)/polyvinyl alcohol (PAA/PVA) nanofibers with nisin (N) from *Lactococcus lactis* showed remarkable antimicrobial activity against *S. aureus*, and interestingly, the antimicrobial effect lasted for 14 days [216]. Taken together, these studies demonstrate that AMPs can be controlled to sustain release directly to the target site for effective therapeutic applications. 

### 4.4. Natural Extracts 

Natural plant or herbal extracts like aloe vera, chamomile, curcumin, propolis, *Biden Pilosa*, *Hibiscus sabdariffa*, *Rosmarinus officinalis*, and *Thymus vulgaris* extracts have been used widely as antimicrobial agents. Due to alkaline stress, these extracts exhibit bactericidal effects through cell membrane hyperpolarisation [217] and cytoplasmic pH change [218]. Bacteria behaviors such as pH homeostasis, membrane transport, motility, resistance, cell division, and electrical communication and signaling depend on the regulation of its membrane potential [219]. The disruption in ion-exchange concentration inside the bacteria membrane can induce its hyperpolarization and cause bacterial structure instability and membrane damage [217,220]. On the other hand, pH and alkaline stress increase metabolic acid production, ATP synthase and change the cell surface properties, leading to bacteria damage and cell death [218].

Recently, PLGA nanofibers fabricated with aloe vera extract having an average diameter of 356 nm with 87.92% porosity were shown to inhibit the growth of *S. aureus* and *S. epidermidis*. No inhibition was observed for pure PLGA nanofibers [221]. A similar finding was reported in which the PCL nanofibers functionalized with chitosan/aloe vera/PEO were found to exhibit antimicrobial activity against *S. aureus* and *E. coli*, as well as promoting rapid proliferation rate for fibroblast cells [222]. An in vivo animal study demonstrated that aloe vera-incorporated nanofibers had accelerated the wound healing and closure of diabetic mice. Overall, nanofibers incorporated with aloe vera extract showed positive effects on antimicrobial activity and rapid wound healing. 

Moringa (MR) extract incorporated into polyacrylonitrile (PAN) nanofibers showed a concentration-dependent antimicrobial activity whereby increased inhibition of *S. aureus* and *E. coli* was observed, as the concentration of extract loaded was increased from 0.1 up to 0.5 g [223]. In another study, MR-chitosan nanoparticles incorporated in gelatin nanofibers were found to inhibit the growth of *Listeria monocytogenes* and *S. aureus* [69]. 

On the other hand, Kegere et al., (2019) analyzed the effect of PVA/chitosan nanofibers blended with Biden Pilosa (BP) crude extract. BP crude extract alone can inhibit 64% of *E. coli* and 51.7% of *S. aureus* growth. In comparison, the fabricated nanofibers showed higher antimicrobial activity with 75.4% *E. coli* and 91% *S. aureus* inhibition [224]. The antimicrobial activity of chitosan is already established [225] and the incorporation of the BP extract into PVA/chitosan nanofibers further enhanced its antimicrobial efficiency. 

## 5. Surface Chemical Functionalization via Monomer Grafting

**Figure 4:**
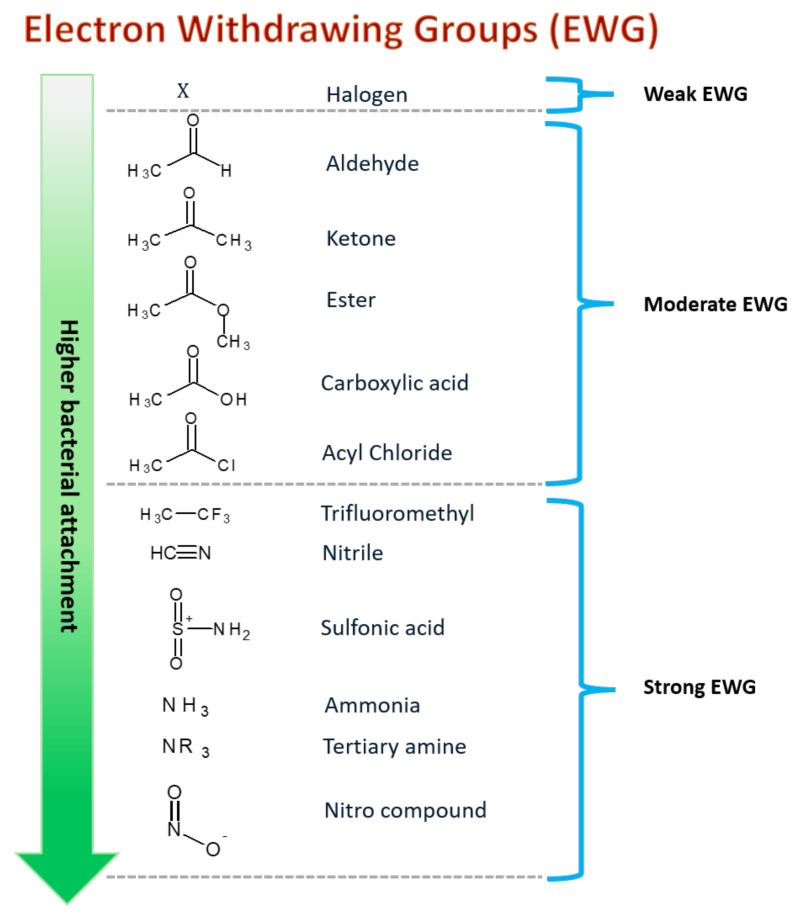
Electron withdrawing group and functional moieties can be functionalized on nanofiber surfaces to improve bacterial adhesion and attachment. The stronger EWGs exhibit higher bacterial attachments.

The surface of nanofibers can be functionalized using different molecular moieties, during or after the treatment electrospinning process [226,227,228]. The three most common methods used to functionalize the surface are the wet chemical method, plasma treatment and graft polymerization [229,230,231]. Post-treatment surface functionalization can also be optimized by adding specific functional groups, mainly electron-withdrawing groups such as carboxylic acids, amines, aldehydes, and acid chlorides (Figure 4) to improve the nanofibers’ surface chemistry such as its wettability, surface charge and surface roughness to further enhance antimicrobial property of the nanofibers [230,232,233,234,235]. 

Abrigo et al., (2015) studied the influence of the fiber wettability, surface charge and surface chemistry of polystyrene (PS) nanofibers functionalized with acrylic acid (ppAAc), allylamine (ppAAm), 1,7-octadiene (ppOct), and 1,8-octadiene (ppCo), using the plasma treatment method, on *E. coli* attachment. The highest amount of *E. coli* attached was observed on the PS surface with ppAAm. Although allylamine is a hydrophilic monomer, its positive charge surface attracts the bacteria and encourages their attachment and proliferation on the surface of nanofibers. Similar to allylamine, acrylic acid is also a hydrophilic monomer. However, only a small proportion of bacteria cells are attached to the ppAAc due to its negatively charged surface. The electrostatic repulsion between the ppAAc nanofibers and *E. coli* resulted in low attachment of bacteria cells.

In contrast, a significant amount of the bacteria was found on the ppOct, attributed to the hydrophobicity of the surface. Although the functionalized nanofibers attracted the bacteria to stick onto the surface, ppAAm and ppOct did not induce bacteria inhibition. Instead, the bacteria proliferated around the nanofibers [158]. The observation indicates that the monomers alone are not enough to kill the bacteria as they do not have antimicrobial properties. 

In another study, upon exposure of PLGA/chitosan nanofibers functionalized with GO-Ag to the attached *E. coli*, *P. aeruginosa* and *S. aureus*, the cells became flattened and wrinkled, causing conformational changes and leading to cell death [198]. The bacterial attachments were also significantly lower when the surface was functionalized with monomers containing cyclic compounds, tertiary butyl, dimethyl hydrocarbon, and a high density of ester groups, due to their rigid structure [236]. In contrast, the attachment can be promoted by functionalizing the surface with monomers containing ethylene glycol and hydroxyl constituents [237]. 

The functionalization of electrospun nanofibers with antimicrobial agents is a promising strategy to combat bacterial infection and resistance. Different functionalization methods and materials used will provide different interactions and mechanism of actions in killing the bacteria. Therefore, the nanofiber criteria and designs discussed above can provide a basic guideline to further understand the relationship between functionalized nanofibers and bacteria cells.

## 6. Conclusions and Future Trends

At present, smart antimicrobial nanofibers have been developed in different fields, including wound dressing, tissue repair and regeneration, nanomedicine, air, and water filtering. Nanomaterial-based antimicrobials can be used as an alternative to antibiotics to achieve an effective therapeutic effect, especially in wound dressing applications. 

The design of fabricated nanofibers plays an important role to ensure antimicrobial effectiveness. Therefore, we have discussed the properties that can influence nanofibers’ bactericidal effects, such as its (1) morphology including size, diameter and porosity, (2) the surface charge of the nanofibers, and (3) surface wettability. However, it has been proven that nanomaterials or nanofibers are not able to fight bacteria alone. The addition of antimicrobial agents is strongly required to enhance the antimicrobial activity of nanofibers. 

Additional studies will be required to enable a deeper understanding on the interactions of these nanomaterials with the target bacteria as bacteria are complex microorganisms that can easily adapt to their surroundings for survival. Additionally, interdisciplinary research involving the chemical, biological, and pharmacological fields is necessary to translate these nanofiber designs clinically.

However, electrospinning is still not ready for the large-scale industrialization of antibacterial fiber production as required from the market. The optimization of nanofibers with highly complex morphologies (i.e., multicomponent, multiaxial fibers) still present some difficulties in terms of large-scale feasibility, and further studies to improve entrapment mechanisms and fabrication processes are needed. 

For this purpose, different manufacturing methods—i.e., the simultaneous or sequential deposition of fibers and/or nanoparticles [238,239] have been optimized to introduce organic or inorganic carriers that provide more appropriate drug release profiles in vitro. In this context, over fiber morphology, drug loading strongly affects the release curve [239]. However, sustained release is strictly conditioned by the polarity of polymer and the drugs (i.e., they have to be similar), and the solubility of the drugs in the polymer solution. In the future, a multidisciplinary approach aimed to design processes and material chemistry could represent a unique route to design innovative carriers with a high degree of morphological and functional complexity, able to control molecular release in a reasonable time to fight bacteria efficiently.

## Figures and Tables

**Table 1:** Key properties of nanofiber scaffolds for antimicrobial applications.

Properties	Effects	References
Nano size	Nanofibers, ranging between 100–1000 nm, are similar to bacteria size, thus can enhance bacterial attachment and inhibition.	[62,63,64,65,66]
Surface area to volume	Nanofibers with smaller diameters provide a higher surface area-to-volume ratio for efficient encapsulation of antimicrobial therapeutic agents.	[67,68,69,70]
High porosity	High porosity allows higher loading of drug or antimicrobial agents into the nanofibers, enhances the surface area, and increases bacteria attachment on the surface of nanofibers.	[71,72,73]
Interconnected pores	Promote oxygen and nutrient exchange, provide structural stability, enhance cell proliferation and ensure sustained release of antimicrobial agents.	[72,74,75,76]

**Table 2:** Electrospun polymer nanofibers loaded antimicrobial agents for effective antimicrobial therapy.

Polymers	Therapeutic Agent	Findings	References
PVA/Pea protein	Cinnamaldehyde	Inhibition of *E. coli* and *S. aureus* increased as the concentration of cinnamaldehyde was increased from 0.5 to 1.5 wt%.	[100]
PCL/ Cellulose acetate	Alkanin and shikonin	Higher drug loaded into the scaffolds (1–5 wt%) inhibited the growth of *S. aureus* and *Staphylococcus epidermidis* and accelerated wound closure.	[28]
PCL/PVA/Pectin	Chelidonium majus L.	The extract was sustained released (65.7%) for up to 30 days and inhibited the growth of *S. aureus* and *Pseudomonas aeruginosa*.	[101]
Chitosan/PEO	Antimicrobial peptides (AMP)	The addition of AMP into the nanofibers enhanced their antimicrobial activity against *E. coli* and *S. aureus*.	[102]
PVA/Collagen	Gentamicin	The release of antibiotic gentamicin can be controlled for up to 72 h.	[103]
PCL/Gelatin	Graphene oxide, tetracycline hydroxide	Nanofibers demonstrated high antimicrobial activity (99%) against *S. aureus* and *E. coli*	[64]
Silk Fibroin	Graphene oxide	Incorporation of graphene oxide reduced the survival rate of *E. coli* and *S. aureus* by 48%.	[29]
PCL/Zein Protein	Tetracycline hydrochloride	Tetracycline was sustained release up to 20 days and the nanofibers inhibited the growth of *S. aureus* and methicillin-resistant *Staphylococcus aureus* (MRSA)	[104]
